# An interprofessional urban health elective focused on the social determinants of health

**Published:** 2018-11-12

**Authors:** Andrew Pinto, Matthew To, Anne Rucchetto, Malika Sharma, Katherine Rouleau

**Affiliations:** 1 Department of Family and Community Medicine, St. Michael’s Hospital, Toronto, Ontario, Canada; 2 Department of Family and Community Medicine, Faculty of Medicine, University of Toronto, Toronto, Ontario, Canada; 3 Dalla Lana School of Public Health, University of Toronto, Toronto, Ontario, Canada; 4 The Upstream Lab, Centre for Urban Health Solutions, Li Ka Shing Knowledge Institute, St. Michael’s Hospital, Toronto, Ontario, Canada; 5 Faculty of Medicine, Dalhousie University, Nova Scotia, Canada; 6 Maple Leaf Medical Clinic, Toronto, Ontario, Canada; 7 Casey House, Toronto, Ontario, Canada

## Abstract

**Background:**

More than half of the world’s population now lives in cities. Health professionals should understand how social factors and processes in urban spaces determine individual and population health. We report on lessons from an interprofessional urban health elective developed to focus on the social determinants of health (SDOH).

**Methods:**

An interprofessional committee developed an urban health elective based in downtown Toronto. Course objectives included promoting collaboration to address SDOH, identifying barriers to care, accessing community-based resources, and learning to advocate at individual- and community-levels.

**Results:**

Seventeen students from eight disciplines participated during the 2011-2012 academic year. Sessions were co-facilitated with community partners and community members identified as experts based on their personal experience. Topics included housing, income and food security, Indigenous communities in urban spaces, and advocacy. Students collaborated on self-directed projects, which ranged from literature reviews to policy briefs for government. Students particularly valued learning about community agencies and hearing from people with lived experience.

**Conclusion:**

The specific health challenges faced in urban settings can benefit from an interprofessional approach informed by the experiences and needs of patient communities. This elective was innovative in engaging students in interprofessional learning on how health and social agencies collaborate to tackle social determinants in urban spaces.

Half of the world now lives in urban settings.^1,2^ The specific needs of vulnerable urban populations remain underrepresented in health training.^3^ We describe a novel interprofessional elective focused on urban health, taught through the lens of the social determinants of health (SDOH).

St. Michael’s Hospital is an inner city hospital in Toronto that trains over 3,000 learners each year from 27 disciplines. An environmental scan found that teaching on urban health at the hospital was limited in most disciplines to a single didactic lecture. An interprofessional course committee was formed with health providers, students, and hospital leadership to design an interprofessional course on urban health.^4^ Course objectives included: 1) promoting interprofessional collaboration; 2) learning about structural barriers and facilitators to attaining “good” health; 3) engaging community-based organizations; and 4) learning appropriate language and skills to advocate for patients. Running during the 2011-2012 academic year, 17 students from eight disciplines (health management, chiropractic studies, dietetic studies, family medicine, nurse practitioner, nursing, pharmacy, and social work) participated in this pilot. Sessions included interactive lectures and experiential learning (Table 1 and Appendix A). Trainees also worked on group projects for presentation at an end-of-year forum.

**Table 1:** Urban health elective curriculum

Session	Description	Community partner(s)
1. Introduction to urban health	Led by the course organizers, and held at St. Michael’s Hospital. This session outlined definitions of urban health and introduced the social determinants of health. A case study of a young woman living with HIV was used to explore how determinants of health, such as race, income, housing status and chronic disease, intersect. Students were introduced to advocacy at individual and community levels. A long-term physician and advocate discussed his experience in calling for improvement in HIV care and care for people with addictions. Lastly, a policy expert introduced a framework for understanding policy change and the role of health professionals in influencing political decision-makers.	The Wellesley Institute, a Toronto based think tank and research organization, with a strong focus on health equity and social determinants.
2. Housing as a determinant of health	This session explored housing as a key determinant of health through a number of causal pathways. Course organizers defined the spectrum of homelessness, and used a case study to examine how illness was related to insecure housing. Students were introduced to individual- and system-level solutions, including Housing First.^22^ The session was held within a large men’s shelter, and included a variety of perspectives. A shelter manager described how it operated and connected with the community, while a person with lived experience of homelessness provided his perspective on the connection between housing and health. Finally a family physician who served as the medical director of a clinic within the shelter, and who had led research studies on homelessness, discussed his experience of introducing managed alcohol programs.^23^ The session ended with a tour of the shelter.	Seaton House, Canada’s largest men’s shelter.
3. Indigenous health in urban environments	This session was held in a community centre that serves Toronto’s Indigenous community. Led by an Indigenous scientist and family physician, this session introduced students to an Indigenous model of health and well-being. The facilitator used her work with urban Indigenous populations to highlight the issue of the relative invisibility of Indigenous communities in urban centers and discussed racism and discrimination experienced by patients in the health system.^24^ The session concluded with a tour of the health centre.	Anishnawbe Health Centre, an Indigenous community health centre.
4. Food and income security as determinants of health	The strong connection between food security and income security was the focus of this session. The session was facilitated by a scientist studying food security, who provided a critique of the traditional charity response to hunger. Her research has exposed the limited role of food banks in solving food insecurity.^25^ The session also featured a family physician with experience in advocacy work around income security. Students worked through a case study highlighting these issues. The session concluded with a tour of the facility, which offers a drop-in, food bank, community cooking, and other services.	The Stop Community Food Centre, a unique organization that evolved from a traditional food bank into a community development hub.
5. Youth and adolescent health in urban environments	The session examined the determinants of health of youth and adolescents. The session was held at Covenant House, a shelter that provides housing and crisis services to youth experiencing homelessness. A unique runaway prevention program, “Reality Check,” was described.^26^ Trainees had an opportunity to tour the shelter.	Covenant House, a large youth and adolescent shelter.
6. Chronic disease management in urban environments	Chronic disease management and the challenges faced by those living in poverty in urban settings was the focus of this session, which took place at St. Michael’s Hospital. The session was led by a pharmacist and a chiropractor, who explored the global burden of non-communicable chronic diseases.^27^ They then used HIV as an exemplar to highlight biological, psychological and social changes that occur with chronic disease. Finally, a clinician-scientist discussed improving care for diabetes patients, and the role of the built environment.^28^ The role of the Community Care Access Centre, which supports the delivery of home care to individuals across Toronto, was discussed.	Toronto Community Care Access Centre, a government funded agency to help patients access home care services
7. Mental health and addictions in urban environments	This session focused on mental health and addictions and was hosted at Toronto Public Health. Speakers included a psychiatry resident physician, a community client of the mental health system, and the manager of a needle exchange program. Finally, a community worker from a community health centre also spoke about hepatitis C prevention and the importance of including harm reduction in any program.	The Works, a harm reduction service and needle exchange run by Toronto Public Health
8. Legal status as a determinant of health	Legal status as a determinant of health was discussed at Romero House, an agency that provides housing, settlement, and advocacy services to refugees. A family physician who directs a large clinic serving refugees discussed clinical care and the role of advocacy. A second family physician, with a focus in women’s health, spoke about her recent advocacy to maintain funding for a bus that brings health services to immigrant and refugee patients. The work of a local advocacy organization, No One is Illegal, was highlighted. The session concluded with a tour of the shelter.	Romero House, a large shelter for refugees, and No One is Illegal, an advocacy organization.

Evaluations for each session measured participant satisfaction of the course and speaker(s) using a scale and open-ended comments. On average, 11/17 evaluations were returned for each session. The average teaching score across 20 different presenters was very high (4.5/5). Students appreciated the mix of theory and practical examples, with one student noting “Good ideas on how to translate ideas into an effective policy or program.” Students identified learning from community agencies and people with lived experience as the best aspects of the course. One student commented, “These clients may have different priorities than what we think they need. It is very important to listen to them and see beyond the medical symptoms.” Students expressed appreciation for new knowledge on SDOH. One student commented: “I learned a lot today that I probably would never have learned if I did not take this elective. It is interesting to see how upstream and downstream thinking can be applied to the discussions today i.e., somehow I feel that the reason why food banks are seen as a solution to hunger is because downstream thinking would identify hunger as the problem and the solution would be to provide food.” Suggestions for improvement included more support for final projects, greater emphasis on case studies and more clinically-oriented experiential learning.

The elective engaged students in immersive education on SDOH.^5,6^ Collaboration with community partners and agencies provided real cases to illustrate patient needs in the urban context.^7,8^ The facilitated visits at community agencies provided students a new perspective on caring for individuals and communities.^9^ Community members and front-line workers led these sessions – discussing experiences of stigma, discrimination and how systems failed – to prevent them from lapsing into “safari tourism,” where privileged health professionals explore exoticized people in poverty.^10^ Consequently, trainees were provided with tools and language for use in advocacy that reflected the input of communities affected.^11^ The positioning of people with lived experience as experts was an essential learning element.^12-15^

This elective was innovative in engaging students in interprofessional learning on how health and social agencies collaborate to tackle social determinants in urban spaces, and introduced trainees to the social causes of health inequities, similar to efforts in social paediatrics.^16-18^ Such curriculum can increase awareness of community resources, challenge assumptions and make the SDOH “real.” As intervening on the SDOH is increasingly embraced,^6,19-21^ health providers will require training on how to develop and implement solutions in close partnership with the people affected by these issues.

## Appendix A Readings and resources

### 1. Introduction and Advocacy

Razack SH. Stealing the pain of others: reflections on Canadian humanitarian responses. Review of Education, Pedagogy and Cultural Studies 2007; 29(4): 375-394.

Navarro V. What we mean by social determinants of health. International Journal of Health Services 2009; 39 (3): 423-441.

Wasylenki DA. Inner city health. CMAJ 2001; 164(2). Available at: http://www.cmaj.ca/content/164/2/214.short

Galea S, Vlahov D. Urban health: evidence, challenges and directions. Ann Rev of Public Health 2005; 26: 341-365. Available at: http://www.annualreviews.org/doi/abs/10.1146/annurev.publhealth.26.021304.144708 (Subscription required)

Bayoumi AM, Hwang S, Silversides A. Inner City Health Research: A Discussion Paper and Summary of the First International Conference on Inner City Health. Submitted to Canadian Institutes for Health Research. Ottawa: CIHR.

Hulchanski D. The three cities within Toronto: Income polarization among Toronto’s neighbourhoods, 1970-2005. Cites Centre, University of Toronto. 2010. Available at: http://www.socialwork.utoronto.ca/Assets/Social+Work+Digital+Assets/Three+Cities+Report.pdf

Toronto Public Health. The unequal city: Income and health inequalities in Toronto, 2008. Toronto: 2008. Available at: http://www.toronto.ca/health/map/pdf/unequalcity_20081016.pdf

### 2. Housing as a determinant of health

Frankish CJ, Hwang SW, Quantz D. Homelessness and Health in Canada: Research Lessons and Priorities. Canadian Journal of Public Health; Mar/Apr 2005; 96: S23-S29. Available at: http://www.raincityhousing.org/docs/cjph.pdf

Power R et al. Health, health promotion and homelessness. BMJ 1999; 318: 590-592

Cheung AM, Hwang SW. Risk of death among homeless women: a cohort study and review of the literature. CMAJ 2004; 170: 1243-7.

Hwang SW. Mortality among men using homeless shelters in Toronto, Ontario. JAMA 2000; 283: 2152-7.

### 3. First Nation/Aboriginal communities in the inner city

Smylie J et al. Our health counts: Urban Aboriginal Health Database Research Project. Community Report: First Nations Adults and Children. CRICH. 2011. http://www.stmichaelshospital.com/crich/our-health-counts-report.php

Anderson M et al. First Nations, Metis, and Inuit Health Indicators in Canada. Background paper. 2006. http://healthcouncilcanada.ca/tree/2.03-BkgrdHealthyCdnsENG.pdf

Ten Fingers K. Rejecting, revitalizing, and reclaiming: First Nations work to set the direction of research and policy development. Canadian Journal of Public Health 2005; 96 (Suppl 1): S60-S63.

Smith LT. Decolonizing Methodologies: Research and Indigenous Peoples. Dunedin, New Zealand: University of Otago Press, 1999. Chapter 1: Imperialism, history, writing and theory.

Stephens C, Porter J, Nettleton C, Willis R. Disappearing, displaced, and undervalued: a call to action for Indigenous health worldwide. Lancet 2006; 367 (9527): 2019–28

### 4. Income security and food security: Making the links

Kirkpatrick SI, Tarasuk V. Food Insecurity and Participation in Community Food Programs among Low-income Toronto Families. Can J Public Health 2009;100(2):135-39.

“Do the Math” tool found at http://dothemath.thestop.org/

Hamelin AM, Beaudry M, Habicht JP. Characterization of household food insecurity in Québec: food and feelings. Soc Sci Med. 2002 Jan;54(1):119-32.

Dachner N, Ricciuto L, Kirkpatrick SI, Tarasuk V. Food purchasing and food insecurity among low-income families in Toronto. Can J Diet Pract Res. 2010 Fall;71(3):e50-6.

Stapleton J. Why is it so tough to get ahead? How our tangled social programs pathologize the transition to self-reliance. Metcalfe Foundation 2007. http://www.metcalffoundation.com/downloads/John%20Stapleton%20-%20why%20is%20it%20so%20tough%20to%20get%20ahead.pdf

Mackenzie H and Shillington J. Canada’s quiet bargain: the benefits of public spending. Canadian Centre for Policy Alternatives 2009. (p. 12-19)

http://www.policyalternatives.ca/~ASSETS/DOCUMENT/National_Office_Pubs/2009/Benefits_From_Public_Spending.pdf

### 5. Youth in the inner city

Kulik DM, Gaetz S, Crowe C, Ford-Jones EL. Homeless youth’s overwhelming health burden: A review of the literature. Paediatric Child Health 2011; 16(6): e43-347.

O’Grady B, Gaetz S, Buccieri K. Can I see your ID? The policing of youth homelessness in Toronto. Homeless Hub Report 5. http://www.homelesshub.ca/ResourceFiles/CanISeeYourID_nov9.pdf

Cameron KN et al. Youth at risk of homelessness in an affluent Toronto suburb. Can J Public Health 2004; 95 (5): 352-356.

Covenant House http://www.covenanthouse.ca/Public/Home.aspx

### 6. Chronic disease management in marginalized populations

MOHLTC. Preventing and managing chronic disease – Ontario’s Framework. 2007. Available at: www.health.gov.on.ca/english/providers/.../pdf/framework_full.pdf

Glazier R, Kennie N, Bajcar J, Wilson K. 2006. Diabetes Care 29(7)1675. A systematic review of interventions to improve diabetes care in socially disadvantaged populations.

Bodenheimer T, Wagner E,. Grumbach K. Improving primary care for patients with chronic illness. 2002. JAMA 266(14)1775.

2008-2013 Action Plan for the Global Strategy for the Prevention and Control of noncommunicable diseases. Available at: http://www.who.int/nmh/publications/9789241597418/en/index.html

### 7. Mental health and addictions

Stergiopoulos, V., Dewa, C., Durbin, J. Chau, N., Svoboda, T. (2010).Assessing the Mental Health Service Needsof the Homeless: A Level-of-Care Approach. Journal of Health Care for the Poor and Underserved 21, 1031–1045.

What is Harm Reduction: A position statement from the International Harm Reduction Association (2010). http://www.ihra.net/files/2010/08/10/Briefing_What_is_HR_English.pdf

Street Health Report (2007). http://www.streethealth.ca/Downloads/SHReport2007.pdf p24-27, p34-36

Hwang, S.W., Colantonio, A., Chiu, S., Tolomiczenko, G., Kiss, A., Cowan, L., Redelmeier, D., Levinson, W. (2008). The effect of traumatic brain injury on the health of homeless people. CMAJ, 179(8), 779-84.

Salyers, M.P. & Tsemberis, S. (2007). ACT and recovery: Integrating evidence-based practice and recovery orientation on Assertive Community Treatment Teams. Community Mental health Journal, 43(6), 619-641.

Strike, C., Leonard, L., Millson, M., Anstice, S., Berkeley, N. & Medd, E. (March 2006). Ontario Needle Exchange Programs: Best Practice Recommendations. http://www.health.gov.on.ca/english/providers/pub/aids/reports/ontario_needle_exchange_programs_best_practices_report.pdf (focus on pages 69-76 – Needle Exchange Best practice Recommendations)

Watson, T.M, Bayoumi, A., Kolla G., Penn, R., Fischer, B., Luce, J. and Strike, C. (2012). Police Perceptions of Supervised Consumption Sites (SCSs): A Qualitative Study. Substance Use & Misuse, 47:364–374.

### 8. Newcomer health and status as a social determinant

Pottie K et all. Evidence-based clinical guidelines for immigrants and refugees. CMAJ 2011; 183 (12). Available at: http://www.cmaj.ca/content/183/12/E824 *** Read sections 1 & 2 (p. 1-11) ***

Toronto Public Health. The global city: Newcomer health in Toronto. November 2011. Available at: http://www.toronto.ca/health/map/pdf/global_city/global_city.pdf

Magalhaes L, Carrasco C, Gastaldo D. Undocumented Migrants in Canada: A Scope Literature Review on Health, Access to Services, and Working Conditions. J Immigrant Minority Health (2010) 12:132–151

Rousseau C, ter Kuile S, Munoz M, Nadeau L, Ouimet MJ, Kirmayer L, Crépeau F. Health care access for refugees and immigrants with precarious status: public health and human right challenges. Can J Public Health. 2008 Jul-Aug;99(4):290-2.

Simich L, Wu F, Nerad S. Status and health security: an exploratory study of irregular immigrants in Toronto. Can J Public Health. 2007 Sep-Oct;98(5):369-73.

Omidvar R and Richmond T. Immigrant Settlement and Social Inclusion in Canada. Laidlaw Foundation 2003. http://www.laidlawfdn.org/cms/file/children/richmond.pdf ***p. 1-19***

Newbold KB. Health care use and the Canadian immigrant population. International Journal of Health Services 2009; 39 (3): 545-565.

Silove S, Steel Z, Mollica RF. Detention of asylum seekers: assault on health, human rights, and social development. Lancet 2001; 357: 1436–37

Canadian Collaboration for Immigrant and Refugee Health http://www.ccirh.uottawa.ca/eng/index.html

No One Is Illegal – Toronto http://toronto.nooneisillegal.org/

No One Is Illegal – Montreal http://nooneisillegal-montreal.blogspot.com/

Health for All http://health4all.ca/

Medical Justice Network – UK http://www.medicaljustice.org.uk/
